# The Importance of Pollinator Generalization and Abundance for the Reproductive Success of a Generalist Plant

**DOI:** 10.1371/journal.pone.0075482

**Published:** 2013-10-07

**Authors:** María Belén Maldonado, Silvia Beatriz Lomáscolo, Diego Pedro Vázquez

**Affiliations:** 1 Instituto Argentino de Investigaciones de las Zonas Áridas, CONICET, CC 507, Mendoza, Argentina; 2 Instituto de Ciencias Básicas, Universidad Nacional de Cuyo, Centro Universitario, Mendoza, Argentina; University of Western Ontario, Canada

## Abstract

Previous studies have examined separately how pollinator generalization and abundance influence plant reproductive success, but none so far has evaluated simultaneously the relative importance of these pollinator attributes. Here we evaluated the extent to which pollinator generalization and abundance influence plant reproductive success per visit and at the population level on a generalist plant, *Opuntia sulphurea* (Cactaceae). We used field experiments and path analysis to evaluate whether the per-visit effect is determined by the pollinator’s degree of generalization, and whether the population level effect (pollinator impact) is determined by the pollinator’s degree of generalization and abundance. Based on the models we tested, we concluded that the per-visit effect of a pollinator on plant reproduction was not determined by the pollinators’ degree of generalization, while the population-level impact of a pollinator on plant reproduction was mainly determined by the pollinators’ degree of generalization. Thus, generalist pollinators have the greatest species impact on pollination and reproductive success of *O. sulphurea.* According to our analysis this greatest impact of generalist pollinators may be partly explained by pollinator abundance. However, as abundance does not suffice as an explanation of pollinator impact, we suggest that vagility, need for resource consumption, and energetic efficiency of generalist pollinators may also contribute to determine a pollinator’s impact on plant reproduction.

## Introduction

Historically, pollination biology was driven mostly by the search of the most efficient pollinator [1] and evidence of specialization and concomitant coevolution [2]. However, more recent evidence indicates that specialization is more often the exception than the rule [3]. Yet, although generalist plants are visited by several pollinator species [3], not all their interactions result in successful pollination events [4]. Moreover, generalist plants have greater capacity for competition, colonization, and invasion than specialists [5]. But is it more advantageous for plants to *interact* with a generalist or a specialist pollinator? Generalist pollinators, i.e., those who visit several plant species, may deposit heterospecific pollen, which usually means loss of pollen and genetic material for the pollen donor, and lower ovule fertilization and seed production for the pollen recipient [6]. In contrast, specialist pollinators are more selective and visit flowers of one or very few plant species, which means lower probability of heterospecific pollen transfer and thus greater per-visit effect, defined here as the number of conspecific pollen grains, pollen tubes and, ultimately, seeds, resulting from one visit by a pollinator to a focal plant.

Besides degree of generalization, pollinator abundance may also be a key factor for plant reproduction [7]. Pollinator impact, the population-level effect of a pollinator population on the reproductive success of a plant, may be more closely related to the pollinator’s interaction frequency, tightly determined by its abundance, than to its per-visit effect [7], [8]. This is because variation in interaction frequency [7] is generally orders of magnitude greater than variation in the per-visit effect between generalist and specialist pollinators [9], [10]. Moreover, frequent pollinator species deposit large amounts of pollen considering all their visits combined, which may result in greater genetic variability of pollen deposited on the stigma; such variability may result in higher quality of seeds due to greater competition among pollen grains for ovule fertilization [11]. In addition, given that generalist pollinators are often more abundant [9], [12], [13] and less fluctuating [14] than specialists, we expect that the pollinator impact [15] of a generalist pollinator on plant reproduction will be greater than that of a specialist.

**Figure 1:**
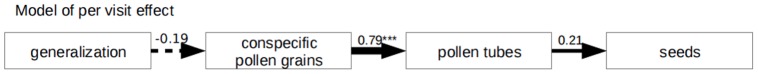
Causal model to evaluate per visit effect on *Opuntia sulphurea*. Arrows represent the causal effect of a variable on another; line width and the number above the arrows represent the magnitude of the pathway coefficient. Continuous lines indicate a positive effect; dashed lines indicate a negative effect. Statistical significance of pathway coefficients is indicated as follows: * = *p*<0.05; ** = *p*<0.01; *** = *p*<0.001. This causal model tests whether pollinator generalization determines the per-visit effect of a specific pollinator species on the focal plant.

**Figure 2:**
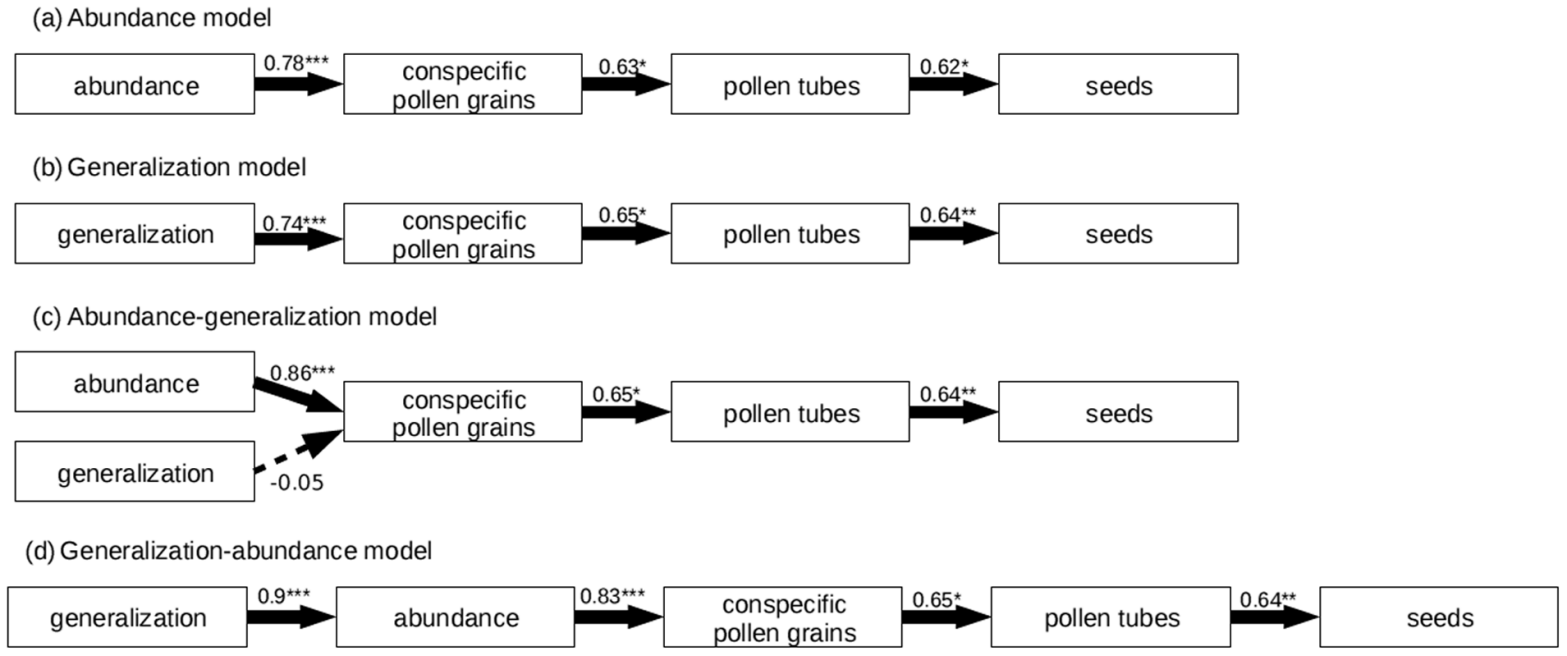
Causal models to evaluate pollinator impact on *Opuntia sulphurea*. Conventions as in Figure 1. The first three models test if pollinator impact on plant reproduction, as estimated by conspecific pollen grains deposited on the stigma, pollen tubes germinated and seeds produced, is determined by pollinator abundance (a), generalization (b), or both (c). Model (d) tests if pollinator generalization determines pollinator abundance and if the latter determines pollinator impact.

Here we analyzed for the first time the relative importance of pollinator generalization and abundance on the reproductive success of a generalist plant. Although previous studies have evaluated separately how pollinator specialization and abundance influence plant reproductive success (e.g. [16]), to our knowledge no study has evaluated simultaneously the relative importance of these pollinator attributes. We address two questions, focusing on a generalist plant, *Opuntia sulphurea* (Cactaceae): (1) does pollinator generalization determine the per-visit effect of pollinator individuals on plants? and (2) do pollinator generalization and abundance determine the impact of pollinator species on plants? For both questions, the effect of a specific pollinator is quantified as the number of conspecific pollen grains deposited on the stigma, the number of pollen tubes germinated, and the number of seeds produced, as a result of a single visit of that pollinator to the plant. For the first question, we hypothesize that generalist pollinators are less effective on a per-visit basis than specialist pollinators because the former may carry heterospecific pollen to the plants they visit (Figure 1). Consequently, we predict that the number of conspecific pollen grains deposited, pollen tubes germinated, and seeds produced per visit will decrease with increasing degree of generalization. For the second question, we hypothesize that pollinator impact on the reproductive success of a plant will be determined by pollinator abundance (Abundance model, Figure 2a), pollinator generalization (Generalization model, Figure 2b), or both (Abundance-generalization model, Figure 2c). Therefore, we predict that overall number of pollen grains deposited, germinated pollen tubes, and seeds produced as a result of the interaction with a specific pollinator will increase with pollinator abundance, pollinator generalization, or both, respectively. Alternatively, we also hypothesize that pollinator generalization determines pollinator abundance, because generalist pollinators can feed on a greater number of resources than specialist pollinators, which may allow generalist pollinators to exist in higher numbers (Generalization-abundance model, Figure 2d). We therefore predict that more generalist pollinators will be more abundant.

## Materials and Methods

### Study System

Our study site is located at Villavicencio Natural Reserve, in the Central Monte Desert in Mendoza, Argentina, at 1.270 m above sea level (32° 31′ S, 68° 56′ W). We worked at a 2-ha shrubland site dominated by *Larrea divaricata* (Zygophyllaceae), *Zuccagnia punctata.*

(Leguminosae), *Condalia microphylla* (Rhamnaceae), *Acantholippia seriphioides* (Verbenaceae), *Opuntia sulphurea* (Cactaceae), *Prosopis flexuosa* (Fabaceae) and *Stypa spp*. (Poaceae) [17], [18]. In our study site most plants flower in spring, between September and December, with a flowering peak in mid October, while some flower in the summer. *Opuntia sulphurea* flowers from early November until mid December. We thank the administration of Villavicencio Natural Reserve and the Direction of Renewable Natural Resources of Mendoza for granting us permission to work in our study site.

The genus *Opuntia* includes 200 species native to the Americas, distributed from southern Canada to Argentina. It is absent in Chile and is scarce in Peru. Several species were introduced in tropical areas of other parts of the world [19]. Their flowers are mostly yellow and occasionally orange in some species, such as *O. maxima*. Flowers are hermaphroditic, actinomorphic, epigean, and rich in pollen and nectar. Vegetative reproduction through cladodes and sexual reproduction are both common in the genus [20]. *Opuntia sulphurea* plants are short, often with creeping cladodes growing in an aligned way. Its flowers are approximately 6 cm long and 5 cm in diameter, with a claviform style 4 to 5 cm long and a stigma with nine thick pistil lobes. The fruit is shaped like a barrel, approximately 5 cm long and 3.5 cm in diameter [19]. Ours is the first study of flower visitors of *O. sulphurea* in the Central Monte Desert in Mendoza.

### Experimental Design

We conducted fieldwork between November 2008 and February 2009. This period covered flowering and fruiting of *O. sulphurea*; such phenological breadth is important to study pollination and reproduction of a plant, because the effect of a pollinator can be different at different phenological stages [21]. To record the effect of pollinators on plant reproduction, we walked randomly in our 1 ha plots searching for *O. sulphurea* plants with flower buttons that seemed ready to open for the first time that same day, based on previous experience with this plant species. All flowers found at this stage were covered with a cloth bag (30 cm×50 cm) to exclude pollinators. We returned to the covered plants often to check whether flowers had opened. Once a flower was open, we removed the bag and observed the flower until the first pollinator visited the plant; if the identity of the flower visitor was unknown, we collected it and took it to a specialist (Table 1). After the first visit, we covered the flower again with the cloth bag to avoid further visits, thereby ensuring that all pollen grains deposited, pollen tubes developed, and seeds produced were a product of one visit by the recorded pollinator (i.e., the per-visit effect). Censuses were conducted simultaneously by two observers, who completed a total of 37 censuses in 37 plants, between 8∶30 and 16∶30, in 6 field days between 24/11/2008 and 18/12/2008, which added up to 40 h of observation. Census length ranged between 10–60 min, starting when the bag was removed and ending whenever the first pollinator came to visit the flower. All recorded visits resulted in deposit of conspecific pollen grains on the flower’s stigma (see below) and, hence, all visitors were considered pollinators.

**Table 1:** Summary data used for analyses.

Floral visitor			Interactionfrequency	Degree ofgeneralization	No. pollen grains	No. pollen tubes	No. seeds
Order	Family	Species					
Hymenoptera	Megachilidae	*Trichoturgus laticeps*	2	1	96.5	53.5	49.5
Hymenoptera	Halictidae	*Augochloropsis* sp.	10	16	68.9	15	14
Hymenoptera	Apidae	*Centris brethesi*	3	7	122	54.3	66.7
Hymenoptera	Andrenidae	*Arhysosage bifasciata*	2	2	36	26	12
Hymenoptera	Megachilidae	*Anthidium* sp.	1	4	299	119	27
Hymenoptera	Apidae	*Bombus opifex*	4	15	94.5	83.5	32
Hymenoptera	Apidae	*Xilocopa atamisquensis*	1	13	13	42	43
Hymenoptera	Apidae	*Svastrides zebra*	1	3	137	23	47
Hymenoptera	Halictidae	*Dialictus* sp.	3	16	*	*	7.5
Diptera	unidentified	unidentified	4		24	25	35.5

Data include number of visits observed, pollinator degree of generalization, and average numbers of pollen grains deposited per visit, pollen tubes developed and seeds produced (* = flowers dried before fruit development). Number of conspecific pollen grains deposited, pollen tubes formed or seeds produced as a result of a single visit of a pollinator species corresponds to the per-visit effect.

We considered three measures of plant reproductive success: number of conspecific pollen grains deposited on the stigma, number of pollen tubes germinated, counted just below the stigma, and number of seeds produced. To ensure fertilization of ovules and subsequent growth of the zygote (i.e., seed production) we allowed 24 to 48 h between flower visitation and pistil removal. In order to visualize and count pollen grains and germinated pollen tubes, all pistils were preserved in 70% ethanol until pollen grains were counted. In the laboratory, pistils were softened with sodium hydroxide (NaOH) 1 N for 24 h at room temperature. They were then washed four times with tap water, and stained with aniline blue dissolved in potassium phosphate (K_2_HPO_4_) 0.15 M for two hours [22]. Using a fluorescence microscope we counted conspecific pollen grains and pollen tubes formed in the stigma and the style. Because pollinator identity was recorded for each visit, the number of conspecific pollen grains and pollen tubes represented measures of the per-visit effect of each pollinator species recorded in the censuses. The number of seeds in a fruit produced by visited flowers was also used as another measure of per-visit effect.

### Estimation of Degree of Generalization of Pollinators

Degree of generalization of pollinators was quantified as the number of plants with which each pollinator species interacts, using data from a four-year study of the whole plant-pollinator interaction network in our study site. These data were collected in 2045 censuses of 5 min each, i.e., a total of 171 hours of observation [23]. We believe that considering all visits as pollination events to estimate the degree of generalization is a reasonable assumption in this case, as Chacoff *et al.* [23] considered only floral visits in which the pollinator contacted the flower’s reproductive organs; furthermore, frequency of interaction was highly and positively correlated with pollinator impact [15]. See Chacoff *et al.* [23] for further details on data collection methods.

### Statistical Analyses

We used path analysis [24] to evaluate our hypotheses, a method that allows the evaluation of causal hypotheses concerning a group of variables (i.e., generalization degree, abundance, and number of conspecific pollen grains, pollen tubes and seeds), assuming that they relate in a linear fashion [24]. This method, which is a type of structural equation modeling (SEM), allows not only to determine how well a model fits the field data, but also to discriminate between alternative models [25].

In our analyses, we considered one model for per-visit effect (Figure 1) and four for population-level effect (species impact) of pollinators (Abundance, Generalization, Abundance-generalization and Generalization-abundance models; Figure 2). The model for per-visit effect included pollinator generalization, and the number of pollen grains, pollen tubes and seeds resulting from a single visit by an individual of a particular pollinator species. The models for population-level effect included pollinator generalization and/or pollinator abundance (estimated based on observed visitation frequency from the 40 h of observation amounted in this study), and the numbers of pollen grains, pollen tubes and seeds resulting from all visits by a pollinator species. The last three variables were calculated as the product of two components, interaction frequency (the total number of visits per pollinator species) and the per-visit effect (explained above). Heterospecific pollen grains deposited were not included in the analysis because they were scarce and presumably did not interfere with reproduction of *O. sulphurea*.

Path coefficients were estimated with the sem function of the sem package of R statistical software. Assessing model fit with the sem function would be problematic because the traditional SEM methods implemented in this function are not appropriate to estimate model fit for small sample sizes such as ours. For this reason, we used a d-separation test to evaluate model fit, a method that is appropriate for data with small samples [26], [27]. The d-separation test considers a basis set, i.e., the *k* pairs of variables that are not directly connected with an arrow in the causal model (Table 2). For each pair of variables *i* in the basis set, it is possible to calculate their probability of independence, *p_i_*, with an appropriate test. We used here the *p*-value associated to Pearson’s correlation test as an estimate of *p_i_*. With this information, we can then calculate the maximum likelihood estimate for each model based on Fisher’s *C* statistic, 
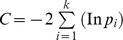
 [26], [27]. Using the *C* value associated to each causal model, we calculated the corrected Akaike’s Information Criterion as AIC_c_ = *C*+2*K* [*n*/(*n*-*K*−1)], where *K* is the total number of free parameters in the model and n is the sample size. The best fitting model was that with the lowest value of AIC_c_. To discriminate among competing models, we used the AIC_c_ difference, ΔAIC_c_, calculated as the difference between a given model and the best-fitting model. Models with ΔAIC_c_<3 cannot be distinguished from the best-fitting model in their fit to the data; in turn, models with 3< ΔAIC_c_<10 can be considered as having considerably less support than the best-fitting model, and models with ΔAIC_c_ >10 can be considered as having essentially no support (Table 2) [28], [29].

**Table 2:** Results of d-separation test.

Models	Basis set of d-separation	*C*	df	*p*	*K*	AIC_c_	ΔAIC_c_
Model per visit	{(generalization, pollen tubes)(generalization, seeds)(conspecific pollen grains, seeds)}	4.1	6	0.34	-	-	-
Models for pollinator impact:							
a-Abundance	{(abundance, pollen tubes)(abundance, seeds)(conspecific pollen grains, seeds)}	21.55	6	0.99	7	91.55	9.6
b-Generalization	{(generalization, pollen tubes)(generalization, seeds)(conspecific pollen grains, seeds)}	11.95	6	0.94	7	81.95	0
c-Abundance-generalization	{(generalization, abundance)(abundance, pollentubes)(abundance, seeds)(generalization,pollen tubes)(generalization, seeds)(conspecific pollen grains, seeds)}	31.65	12	1	8	191.65	109.7
d-Generalization-abundance	{(generalization, conspecific pollen grains)(generalization, pollen tubes)(generalization, seeds) (conspecific pollen grains, seeds)(abundance, pollen tubes)(abundance, seeds)}	30.89	12	1	9	228.98	147.03

For each model, the basis set lists the statistically testable predictions of independence made by each model. Also given is Fisher’s C statistic and the associated *p*-value for each path model. Non-significant C values suggest that we can accept the proposed model. *K* is the total number of free parameters in a model. AIC_c_ is Akaike’s information criterion of model selection and ∶AIC_c_ is the relative difference in AIC_c_ between a given model and the best-fitting model (b-Generalization).

All analyses were done with R statistical software, version 2.9.1 [30]. Path analysis and structural equation modeling were conducted with the sem package in R [31].

## Results

We observed 31 visits by 10 flower visitor taxa, with a mean of 5 visits and 3 visitor species per day. We recorded pollen grains and pollen tubes in all visited pistils, while 75% of visited flowers produced seeds. At the per visit level, the proposed model had a good fit to the data (Table 2). According to this model, degree of generalization of pollinators had a negative effect on plant reproduction at the per-visit level (Figure 1). However, this effect was weak and statistically non-significant. Conspecific pollen deposited was positively and significantly related to pollen tube development, while the latter was weakly and non-significantly related to seed production (Figure 1).

At the population level, all models passed the d-separation test and thus provided reasonable fits to the data (Table 2). Both abundance and generalization had significant effects on conspecific pollen grains, suggesting that both contribute to determine the species impact of pollinators. When confronted with AIC, the best-fitting model was the Generalization model (Figure 2b). In this model, pollinator generalization had a significantly positive effect on number of pollen grains deposited, of the latter on pollen tubes developed in the stigma, and of the latter on the number of seeds produced.

## Discussion

Our results suggest that the per-visit effect of a pollinator on *O. sulphurea*’s fitness could be independent of whether a specific pollinator is a generalist or a specialist. Although this result cannot be generalized to all plant species (as specialist pollinators may indeed be more effective than generalists for some plant species [16]), our results support the notion that pollinator specialization is not essential for effective pollination. At the same time, it is important to note that all pollinator species may have behaved effectively as specialists during our study, as we found little heterospecific pollen in the stigmas of *O. sulphurea*, which suggests that they visited almost exclusively flowers of this species. A plausible explanation for this apparent specialization is pollinator constancy, whereby even the most generalized pollinators may tend to visit flowers of the same species in a given foraging bout, which helps the insect to locate flowers efficiently [32], hence decreasing interspecific pollen transfer.

When focused at the population level, our results suggest that generalist and abundant pollinators have the greatest effect on *O. sulphurea*’s fitness, as shown by the significant path coefficients between these pollinator attributes and conspecific pollen grains in Figure 2a,b. However, when we considered pollinator abundance and generalization in a set of competing causal models, we found that the model that included generalization but not abundance had the best fit to the data. Thus, generalist pollinators have the greatest species impact on pollination and reproductive success of *O. sulphurea*.

Why should generalist pollinators have the greatest impact? As we conjectured in the Introduction, this effect may be partly explained by pollinator abundance as, even though the causal models including abundance were not the best-fitting ones, abundance did have a significant, positive effect in the model that included it as a predictor of conspecific pollen grains (Figure 2a); in addition, pollinator generalization and abundance were positively correlated (Spearman’s *r* = 0.58), which indicates that generalist pollinators also tend to be abundant. However, abundance does not suffice as an explanation of pollination and reproductive success of *O. sulphurea* because, as we said, it was not included in the best-fitting model. An alternative explanation of why generalist pollinators may have greater impacts is that they may be more vagile than specialist pollinators because of a greater need for resource consumption resulting from their lower energetic efficiency [33]; thus, all else being equal, a generalist pollinator would contribute more to pollination because it would visit more flowers than a specialist pollinator.

It is important to acknowledge that our results are based on a rather limited sample size (31 pollinator visits) in spite of substantial sampling effort (40 h of observation). Although a larger sample size would certainly be desirable, we believe that increasing the sample size would be unlikely to affect our conclusions, as the d-separation test used in our analysis is robust to small sample sizes, and all relationships in our best-fitting model were statistically significant.

We close with three suggestions for potential avenues for future research. First, most studies of multi-species interactions (including ours) have used population averages of pollinator and plant attributes (such as pollinator abundance and generalization, and plant reproductive success), thus ignoring individual variation within populations. Such population-level aggregation may influence inferences on ecological processes [34]. Thus, it is important that future studies of the role of pollinator abundance and specialization on plant reproduction consider inter-individual variation in these population attributes. Second, a measure of seed viability would offer a more accurate measure of plant fitness, because some of the visits we recorded may have transferred geitonogamic pollen and, hence, might potentially yield lower quality seeds. Third, all available evidence for the role of pollinator abundance for plant reproduction comes from short term studies (again, including our own study). A narrow temporal scale may not allow inferences about the long term consequences of plant-pollinator interactions. For example, short term studies cannot assess how temporal fluctuations in pollinator abundance may affect their effect on plant fitness. Long-term studies on plant-pollinator interactions, together with detailed knowledge of the effect of specific pollinators with differing abundance and specialization, such as that yielded by this study, will allow us to understand the microevolutionary processes in mutualisms that are responsible for the maintenance and generation of biodiversity.
